# Hybrid Porous Microparticles Based on a Single Organosilica Cyclophosphazene Precursor

**DOI:** 10.3390/ijms21228552

**Published:** 2020-11-13

**Authors:** Vanessa Poscher, George S. Pappas, Oliver Brüggemann, Ian Teasdale, Yolanda Salinas

**Affiliations:** 1 Institute of Polymer Chemistry, Johannes Kepler University at Linz, Altenberger Strasse 69, 4040 Linz, Austria; vanessa.poscher@jku.at (V.P.); georgios.pappas@jku.at (G.S.P.); oliver.brueggemann@jku.at (O.B.); ian.teasdale@jku.at (I.T.); 2 Linz Institute of Technology (LIT), Johannes Kepler University at Linz, Altenberger Strasse 69, 4040 Linz, Austria

**Keywords:** porous organosilica microparticles, cyclophosphazenes, post-functionalization, degradability, hybrid materials

## Abstract

Porous organosilica microparticles consisting of silane-derived cyclophosphazene bridges were synthesized by a surfactant-mediated sol-gel process. Starting from the substitution of hexachlorocyclotriphosphazene with allylamine, two different precursors were obtained by anchoring three or six alkoxysilane units, via a thiol-ene photoaddition reaction. In both cases, spherical, microparticles (size average of ca. 1000 nm) with large pores were obtained, confirmed by both, scanning and transmission electron microscopy. Particles synthesized using the partially functionalized precursor containing free vinyl groups were further functionalized with a thiol-containing molecule. While most other reported mesoporous organosilica particles are essentially hybrids with tetraethyl orthosilicate (TEOS), a unique feature of these particles is that structural control is achieved by exclusively using organosilane precursors. This allows an increase in the proportion of the co-components and could springboard these novel phosphorus-containing organosilica microparticles for different areas of technology.

## 1. Introduction

Silica-based materials have become one of the most intensively investigated tools for advancing a wide range of chemical technologies, such as catalysis, sensing, as coatings, for purification or adsorption of hazardous pollutants, as well as for drug-controlled release [1]. The reason for this is that they are inexpensive and scalable and their chief physicochemical properties are very appealing: mechanical and chemical stability, easy controllable particle size, well-defined surface area, as well as high Si-OH group content, which allows for (multi)functional surface modification with many different interesting chemical moieties, many of which are commercially readily available. In spite of their wide range of applications, more sophisticated formulations are required, for example towards evolved sustainable, biodegradable solutions [2].

Porous and non-porous silica particles can be readily prepared from a silicon alkoxide precursor via the well-known sol-gel route [3,4]. This reaction is performed under controlled conditions, in alcohol solution, catalyzed by a base, following simultaneous hydrolysis and polycondensation of the silicon alkoxide molecules to result in the formation of Si–O–Si network. The subsequent growth and formation of a porous network of silica particles is mediated by the use of a specific surfactant during synthesis, which acts as a structure directing agent [5]. The porosity of these silica particles gives these materials high surface areas and makes them extremely useful for enhanced controlled drug delivery applications [6] and for catalysis [7]. Traditionally, tetraethyl orthosilicate (TEOS) is employed as precursor for the preparation of mesoporous silica nanoparticles (MSN) [8]. The silica bridge, and consequently the porous structure, can be modified by mixing TEOS with other organosilane precursors. Nevertheless, when the reaction is performed exclusively with organosilane precursors, then periodic mesoporous organosilicas (PMOs) are obtained [9,10]. PMOs have attracted much attention since their mesoporous nature is combined advantageously with an organic-inorganic fusion within the silica walls. The location of different types of functional groups within the channel walls bridging the silicon centers opens many pathways towards tailored porous silica micro/nanoparticles [11]. Interestingly, the incorporation of emerging potentially degradable units while keeping stable the silica framework may drive towards future promising purposes, such as degradable drug delivery carriers. However, the very high organic content, which leads to many of the most interesting possibilities for PMOs, also makes them challenging to prepare. Larger size of the bridge in organo-bridged alkoxysilanes, in comparison to the short alkoxide precursor TEOS, often causes difficulties in the co-assembly of the surfactant micelle and the silane source. That is reflected in the very few examples reported in literature where PMOs are exclusively synthesized with organosilica-bridged units, as opposed to mixtures with TEOS [12].

Organophosphazenes are well-studied hybrid organic–inorganic molecules with a characteristic structure of alternating phosphorus and nitrogen atoms, the former bearing two organic substituents [13]. A wide range of organic molecules can be utilized as substituents and they have a decisive impact on the material characteristics, hence can be used to tailor the properties towards the final requirements. Some poly(organo)phosphazenes undergo hydrolytic degradation in ambient conditions to non-toxic degradation products, which has formed the basis of their recent development in a number of medical applications [14,15,16]. The most frequently used precursor is the six-membered ring, which can also be used as a building block for complex architectures such as dendrimers [17] and cyclomatrix materials [18]. For the preparation of cyclotriphosphazene-containing materials, hexachlorocyclotriphosphazene is commonly used as starting precursor, followed by the substitution of chlorine atoms using (mostly) organic side groups. Moreover, cyclotriphosphazenes can be functionalized for example with various alkoxysilanes to increase their introduction in the sol-gel process [19,20,21], particularly, for various promising applications as bio-applied dendrimers [17], in photoelectronics [22] and as flame retardants [23].

Based on our previous experience in preparing compact organosilica phosphazene-based hybrid nanoparticles [24] and our current interest in porous silica-based hybrid materials, we describe herein the synthesis of novel porous organosilica hybrid microparticles from a single silane-derived cyclophosphazene precursor through the surfactant-mediated sol-gel process. We report the morphology along with their potential functionalization and hydrolytic degradation behavior.

## 2. Materials and Methods

### 2.1. Chemicals

Hexachlorocyclotriphosphazene (98%) was purchased from Acros organics; 3-mercaptopropionic acid (99%) and allylamine (>98%) from Alfa Aesar (Karlsruhe, Germany); 2,2-dimethoxy-2-phenylacetophenone (DMPA) (99%) and (3-mercaptopropyl)trimethoxysilane (95%) were obtained from Sigma-Aldrich (Vienna, Austria); anhydrous THF, trimethylamine and cetyltrimethylammonium bromide (CTAB) (≥99%) from VWR (Vienna, Austria); ethanol (99%) from Fluorochem (Derbyshire, UK); and NaOH (98%) from J. T. Baker (Fisher Scientific, Wien, Austria). Deuterated chloroform and methanol for NMR experiments from Eurisotop (Saint Aubin, France). Triethylamine was distilled before usage for the substitution reaction. All other chemicals and solvents were used as received.

### 2.2. Characterization Methods

For measuring ^1^H, ^31^P, ^13^C NMR spectra a Bruker Avance III 300 was utilized (Bruker BioSpin GmbH, Rheinstetten, Germany). The ^1^H NMR spectra were taken at 300 MHz using CDCl_3_ or MeOD as solvent. The ^31^P NMR spectra were measured at 121 MHz. The ^13^C NMR spectra were conducted at 75.47 MHz. For Fourier-transform infrared spectroscopy (FT-IR) a Spectrum 100 FTIR spectrometer (PerkinElmer, Buckinghamshire, UK) was utilized with attenuated total reflection (ATR), and the measurements were performed in the range from 600 to 4000 cm^−1^. N_2_ adsorption-desorption isotherms were recorded on a Micromeritics TriStar II 3020 surface area and porosimeter analyzer (Micromeritics, Norcross, GA, USA). At 77.30 K ca. 40 measurement points were taken. Before measurement, the samples were degassed at 80 °C for 12 h. On a Zetasizer nano ZSP, (from Malvern, Worcestershire, UK), DLS measurements were carried out, in 10 mM NaCl solution as dispersant (1 mg mL^−1^) in disposable cuvettes (DTS 0012), previously sonicated for 1 h. The DLS measurements via intensity were performed at 25 °C without filtration before the measurements. TGA measurements were performed on a Q5000 TA (TA instruments, New Castle, DE, USA) under nitrogen atmosphere (25 mL min^−1^) in a platinum pan with a heating program from 40 to 900 °C at 10 °C min^−1^. SEM images were obtained with Jeol 6400 (Jeol, Peabody, MA, USA). TEM images and elemental mapping analysis (TEM-EDS) were obtained with Jeol JEM-2200FS microscope (Jeol, Peabody, MA, USA).

### 2.3. Synthesis of Silane Derived Cyclic Phosphazenes SiCPz1 and SiCPz2

Allylamine substituted cyclic phosphazene CPz was synthesized according to literature procedures [24]. Allylamine-substituted phosphazene (0.5 g, 1.06 mmol) and 2,2-dimethoxy-2-phenylacetophenone (DMPA) (80.5 mg, 0.31 mmol) were dissolved in 13.32 mL ethanol. The solution was degassed for 1 h with nitrogen, and (3-mercaptopropyl)trimethoxysilane was added to the solution, 1.18 mL (6.35 mmol, 6 eq.) to yield to SiCPz1 (fully substituted double bonds) or (0.59 mL, 3.18 mmol, 3 eq.) yielding to SiCPz2 (half substituted double bonds). ^1^H NMR (SiCPz1, 300 MHz, CDCl3, δ/ppm): 3.54 (s, 54H); 2.98 (broad s, 12H); 2.52–2.42 (m, 24H); 1.72–1.56 (m, 24H); 0.70–0.65 (t, 12H); ^31^P NMR (SiCP1z, 300 MHz, CDCl_3_, δ/ppm): 16.80; ^13^C NMR (SiCPz1, 300 MHz, CDCl_3_, δ/ppm): 50.43; 39.92; 34.98; 31.40; 29.19; 22.89; 8.54; ^1^H NMR (SiCPz2, 300 MHz, CDCl_3_, δ/ppm): 5.91–5.80 (m, 3H); 5.20–5.14 (d, 3H); 5.02–4.98 (d, 3H); 3.51 (s, 33H); 2.95 (broad s, 6H); 2.51–2.44 (m, 12H); 1.74–1.60 (m, 12H); 0.72–0.66 (t, 6H); ^31^P NMR (SiCPz2, 300 MHz, CDCl_3_, δ/ppm): 17.30; ^13^C NMR (SiCPz2, 300 MHz, CDCl_3_, δ/ppm): 137.18; 114.58; 50.38; 43.43; 39.83; 34.91; 31.27; 29.10; 22.81; 8.48 (see all spectra, including the heteronuclear single quantum coherence (HSQC) spectrum of both precursors, in Figures S1–S6).

### 2.4. Synthesis of Porous Organosilica Phosphazene Based Microparticles SiCPz1-PM1 and SiCPz2-PM2

A mixture of cetyltrimethylammonium bromide (CTAB) (338.6 mg, 0.93 mmol), 0.59 mL 2 M sodium hydroxide (NaOH) solution, and 81 mL Milli-Q water was stirred for 1 h at 80 °C. After micelle formation, the silane source (1.063 mL phosphazene precursor solution in EtOH, 0.085 mmol) was added dropwise. A white precipitate formed and the solution was stirred for 2 h at 80 °C. Afterwards the mixture was cooled to room temperature while stirring, the particles were obtained by centrifugation for 15 min at 5000 rpm and washed with Milli-Q water until they were neutral, ethanol (EtOH) was used for the last washing step. The particles were dried resulting in a beige fine powder (around 30 mg of SiCPz1-PM1 and around 12 mg of SiCPz2-PM2 were obtained). Both microparticles were characterized by standard techniques used as in traditional MSNs synthesis.

### 2.5. Hydrolytic Degradation Studies of the Porous SiCPz1-PM1 Microparticles

The SiCPz1-PM1 microparticles containing fully substituted phosphazene precursor were studied under different hydrolytic conditions and time. A total of 1 mg of organosilica microparticles were suspended in aqueous solution (1 mL) at both acidic pH 2.0 and neutral pH 7.0. The mixture was continuously stirred at room temperature for 4 weeks. After 1 week and after 4 weeks, the particles were collected by centrifugation, washed extensively with Milli-Q water until neutral pH, and dried overnight. For transmission electron microscopy (TEM) measurements, the particles were resuspended in EtOH (1 mL) and ultrasonicated for 30 min before deposition on the copper grids.

### 2.6. Post-Functionalization of the Porous SiCPz2-PM2 Microparticles

The SiCPz2-PM2 microparticles containing the partially substituted phosphazene precursor SiCPz2 were afterwards functionalized with 3-mercaptopropionic acid through thiol-ene photoreaction. For this, 10 mg of microparticles SiCPz2-PM2 and photoinitiator 2,2-dimethoxy-2-phenylacetophenone (DMPA) (5 mg, 0.0195 mmol) were stirred in 10 mL EtOH. After complete dissolution of the photoinitiator, the mixture was degassed with nitrogen for 60 min. A total of 1 mL of 3-mercaptopropionic acid, was injected through a septum and again, a thiol-ene photoreaction was performed. After the UV reaction, the mixture was centrifuged to collect the particles and washed with copious amounts of water until no more well-recognizable thiol odor was detected. The functionalized SiCPz2-PM2 microparticles were characterized using Fourier-transform infrared spectroscopy (FT-IR) and thermogravimetric analysis (TGA).

## 3. Results and Discussion

### 3.1. Synthesis and Characterization of the Cyclic Organosilica Phosphazenes Bridges

**Figure 1:**
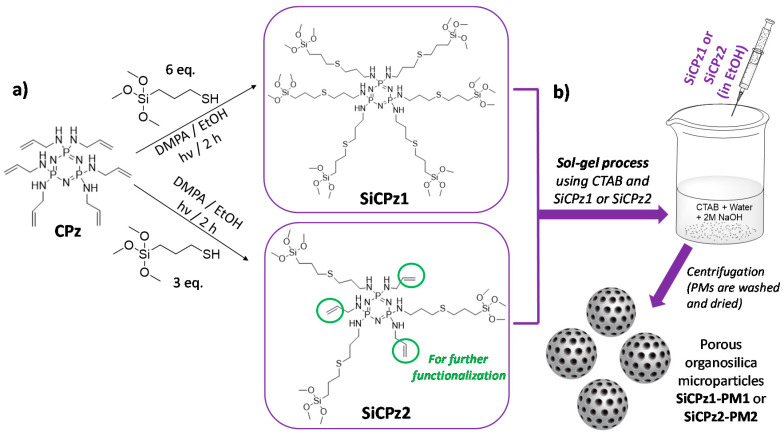
Scheme of (**a**) synthesis of organosilica phosphazene bridges (fully substituted CPz1 precursor yielding to SiCPz1, or half substituted yielding to SiCPz2, able to be further functionalized), both used for the (**b**) preparation of hybrid porous organosilica microparticles (SiCPz1-PM1 and SiCPz2-PM2) following well-known CTAB mediated sol-gel process, collected by centrifugation.

Cyclophosphazene-based silica hybrid nanoparticles were prepared by a surfactant-mediated sol-gel method, as shown in Figure 1b. Due to the common crosslinking tendency of the silane-containing moieties, the starting precursor was prepared using a two-step method. Initially, a stable allyl-substituted cyclic phosphazene trimer (CPz) was prepared via a nucleophilic substitution reaction of hexachlorocyclotriphosphazene with allylamine [25]. The precursor CPz containing six double bonds was obtained with 83% yield after purification, characterized before further functionalization with alkoxysilanes. The success of the substitution reaction was clearly shown by the corresponding ^1^H, ^31^P, and ^13^C nuclear magnetic resonance (NMR) spectra (see experimental details).

The ^31^P NMR spectrum showed a single peak at 18.75 ppm indicative for complete substitution of all chlorine atoms. This allylamine substituted precursor is stable and can be stored over a long period of time. To attach the silane groups necessary for silica particles preparation, a thiol-ene photoreaction was performed to inset the silane groups containing (3-mercaptopropyl)trimethoxy silanes to the double bonds [21,24]. The photochemical reaction is fast and quantitative, hence the product can be used directly for the particle synthesis. This two-step approach is advantageous to the direct substitution of the phosphazene with, for example (3-aminopropyl)triethoxysilane, which requires tricky purification and storage of this hydrolytically sensitive precursor [26].

**Figure 2:**
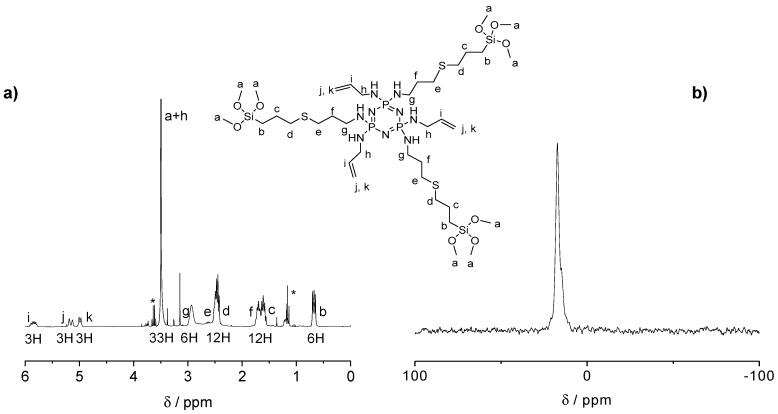
(**a**) ^1^H NMR spectrum and (**b**) ^31^P NMR spectra of the half-substituted precursor SiCPz2 in CDCl_3_ (traces of EtOH marked with *).

The influence of the number of silane groups on the formation of porous microparticles was investigated by preparing two types of organosilica bridge units (see Figure 1a). Six or three equivalents (eq) of (3-mercaptopropyl)trimethoxysilane were added to the double bonds of previously prepared CPz, leading to SiCPz1 and SiCPz2, respectively (reaction adapted from [27]). The precursor molecules SiCPz1-2 were characterized and stored in an ethanolic solution under inert conditions. ^1^H, ^31^P, and ^13^C NMR, together with FT-IR measurements were utilized to confirm the linkage of the silane groups to CPz trimer. From the ^1^H NMR spectra of the molecules SiCPz1 and SiCPz2 (Figure S1 and Figure 2a, respectively), the large singlet at 3.54 or 3.51 ppm can be clearly assigned to the 54 and 27 protons from the attached trimethoxysilane (–Si–O–CH_3_) groups. Due to the high tendency of this groups towards hydrolysis, the expected number of protons differed slightly from the measured protons with NMR spectroscopy. In fact, these functional groups are susceptible to hydrolysis even in the smallest traces. Moreover, in the case of SiCPz2 visible peaks between 5 and 6 ppm were assigned to the double bonds from the allylamine units (see Figure 2a).

In addition, ^13^C NMR spectra showed the presence of 7 or 10 carbon atoms related to half-substituted SiCPz2 or fully silane derived phosphazenes SiCPz1, respectively (see Figures S3 and S4). To prevent undesired crosslinking reactions and the formation of Si–O–Si bonds, the solvent was not removed completely, thus visible in the NMR spectrum. The ^31^P NMR spectra only showed one peak at 16.80 and 17.30 ppm (after the UV reaction) for SiCPz1 and SiCPz2, respectively (see Figure S2 and Figure 2b, respectively). Furthermore, to assign all protons and carbons before using these phosphazene-based silica source for the microparticles preparation, 2D NMR measurements were performed. The heteronuclear single quantum coherence (HSQC) spectrum of precursors SiCPz1 and SiCPz2 (Figures S5 and S6) allowed the assignment of directly linked carbons and protons.

FT-IR measurements were also carried out to confirm the functional groups present in the precursor solutions SiCPz1-2 (see comparison spectra in Figure S7). The fully substituted SiCPz1 showed strong bands at 1072 cm^−1^ from Si–O–C asymmetric stretch and at 1244 cm^−1^ assigned to the endocyclic P-N bond vibration of the phosphazene trimer. Bands at 2944 and 2832 cm^−1^ were characteristic for C–H groups from the precursor solution, whereas bands observed at 798 cm^−1^ and 663 cm^−1^ related to –Si–O–C and C–S–C vibration respectively, were clear indications of the covalently attached alkoxysilanes. C=C groups from the free allylamine were also observed on the spectrum of half-substituted precursor SiCPz2. However, bands at 953 cm^−1^ assigned to –Si–OH, were related to possible silane hydrolysis and even undesired crosslinking in the precursor solution.

### 3.2. Synthesis of Porous Organosilica Phosphazene-Based Microparticles

For the synthesis of novel hybrid porous organosilica phosphazene microparticles, both phosphazene precursors were utilized (adapted from [28]) in order to study the effect on particles and pores formation. The optimal reaction conditions to obtain spherical and porous particles, by using the phosphazene-based organosilica source as a single component, were extensively studied (see experimental details). In the optimized conditions, an ethanolic solution of SiCPz1-2 was directly added dropwise to a mixture of surfactant CTAB/water/sodium hydroxide, and sol-gel reaction was performed at 80 °C for 2 h. A white precipitate was formed immediately after addition of the precursor solution, possibly due to the fast particle’s formation (see Figure 1b). The particles were collected and washed by centrifugation using Milli-Q water until neutral pH was obtained. A final drying step resulted in beige fine powder (see Figure 4, inset), yielding into SiCPz1-PM1 and SiCPz2-PM2, respectively. The surfactant was subsequently removed by ionic exchange, following methods adapted from the literature [9]. For this, the particles were stirred under refluxing conditions in an ethanolic sodium chloride (NaCl) solution, and the complete surfactant extraction was followed by thermogravimetric analysis and FT-IR spectroscopy (see Figure S9 and Figure 4, respectively).

### 3.3. Characterization of the Prepared Porous Organosilica Microparticles

**Figure 3:**
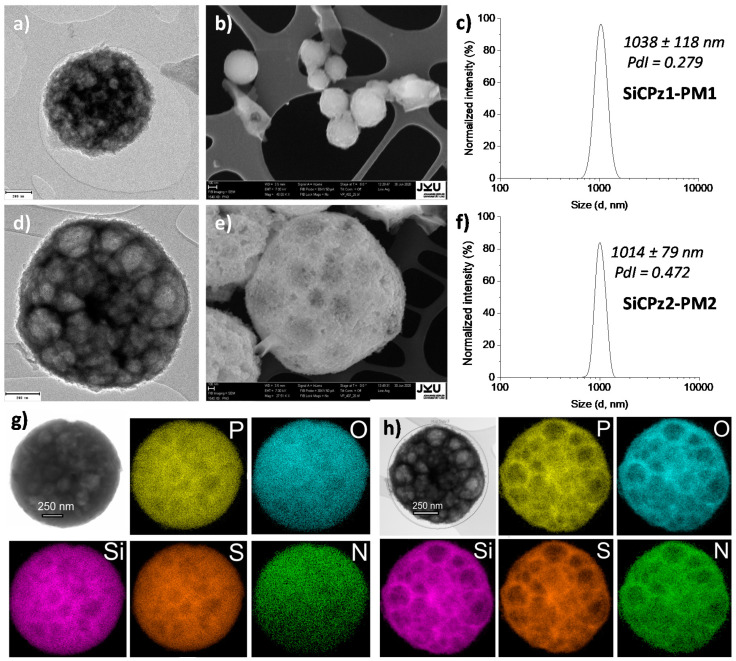
(**a**) TEM, SEM, and DLS measurements showing the morphology features of (**a**–**c**) porous organosilica microparticles SiCPz1-PM1 and (**d**–**f**) SiCPz2-PM2 (TEM and SEM scales 200 and 100 nm, respectively); (**g**,**h**) TEM-EDS elemental mapping (at %) images of (**a**) SiCPz1-PM1 and (**b**) SiCPz2-PM2, respectively (showing main elements P, O, Si, S, and N), scale = 250 nm.

Transmission electron microscopy (TEM) and scanning electron microscopy (SEM) were utilized to confirm the morphology (sizes of particles and pores) of the synthesized silica-phosphazene hybrid particles. Using both silane-derived precursors, TEM measurements showed similar formation of spherical particles with average diameter of 1000 nm, in agreement with hydrodynamic diameters obtained from dynamic light scattering (DLS) measurements (see SiCPz1-PM1 in Figure 3a–c and SiCPz2-PM2 in Figure 3d–f). Interestingly, SiCPz1-PM1 were slightly smaller than those synthesized with the half-substituted precursor solution, however more homogeneous size distribution was observed in the TEM images. This effect may be attributed to the larger amount of reactive silane groups, leading to an initial faster particle formation and thus to a more homogeneous size distribution. Hybrid microparticles with large pores (from around 70 to 250 nm) were obtained, as measured from the TEM images (Figure 3a,d), also confirmed by SEM (Figure 3b,e).

N_2_ adsorption–desorption measurements are traditionally used to determine pore size and volume in MSNs. In our case, the pores were clearly observed and measured by using TEM images, even in the isotherms of the SiCPz2-PM2, a step in the desorption curve between 0.4 and 0.5 (P/P_0_) was observed (see Figure S8), characteristic for porous materials. Interestingly, a notable increase in volume of gas adsorbed after the surfactant removal was as well observed. Nevertheless, the gas used for the adsorption–desorption measurements is commonly nitrogen, not very suitable to measure large pores (>50 nm). Hence, the data obtained via this technique were not further considered.

In addition, the composition of the porous microparticles was confirmed semiquantitatively by energy dispersive X-ray spectroscopy (EDS). All the expected elements O, S, Si, P, and N were clearly shown to be uniformly distributed over the whole microparticle (mapping analysis in Figure 3g,h). It was not possible to confirm the amount of carbon with this measurement, because the TEM sample holder film also consisted of carbon, which will lead to miscalculations. At the same time, Cu was also detected because of the TEM grids. Traces of Br were assigned to possible residues of surfactant.

**Figure 4:**
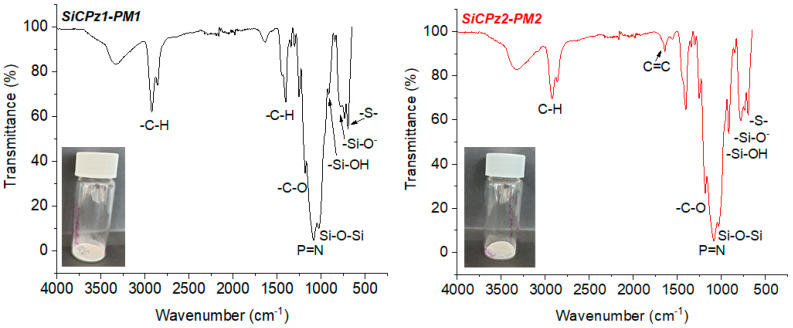
FT-IR spectra of porous microparticles SiCPz1-PM and SiCPz2-PM after surfactant removal, inset: powder-like microparticles image.

Furthermore, FT-IR measurements added information about the chemical structure of both organosilica microparticles, SiCPz2-PM1 and SiCPz2-PM2 (see Figure 4). The asymmetric stretch vibration of Si–O–Si bridge and the P=N group appeared and overlapped within the broad peak between 1020 and 1260 cm^−1^ while the peaks at 1640 and 913 cm^−1^ were assigned to –SiOH. The FT-IR spectra of both samples were similar, however, the spectrum of SiCPz2-PM2 showed a sharper peak at 1640 cm^−1^, characteristic of the alkene stretch vibration (C=C). The peak at 685 cm^−1^ was attributed to the C–S–C vibration, which appeared slightly more intense in SiCPz2-PM1 due to the larger number of sulphide groups compared to SiCPz2-PM2. The two intense peaks between 3200 and 2800 cm^−1^ were an indication of traces of surfactant still encapsulated into the pores. These peaks are characteristic for the symmetric and asymmetric stretching vibration of the methylene chain, also commonly used as a sign for completed surfactant removal, since that characteristic peak may decrease in size, but it did not disappear completely due to the presence of –CH groups from the allylamine groups in the phosphazene precursor molecule (see Figure S9).

### 3.4. Hydrolytic Degradation Studies of SiCPz1-PM1 Microparticles

**Figure 5:**
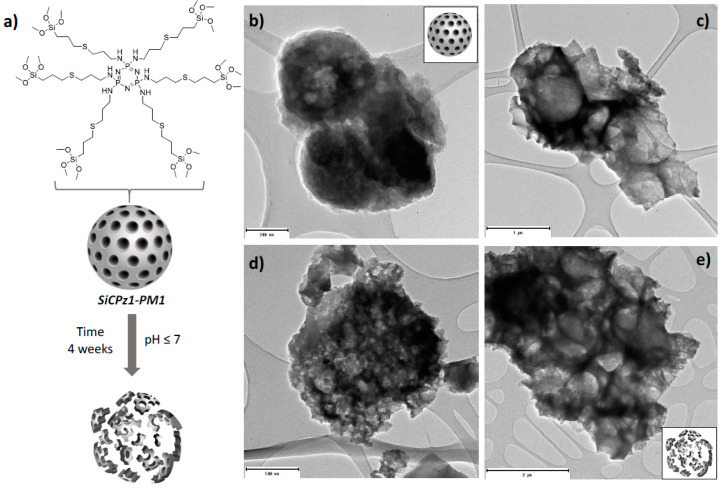
Illustration of the SiCPz1-PM1 degradation (**a**) and TEM images of the SiCPz1-PM1 in an aqueous solution at neutral pH 7 for 1 (**b**) and 4 weeks (**c**) and at acidic pH 2 for 1 (**d**) and 4 weeks (**e**). Scales: 200, 500, 1000, and 2000 nm.

Preliminary hydrolytic degradation studies were carried out under different conditions on SiCPz1-PM1 microparticles. The particles were stirred at pH 2 and pH 7 for 4 weeks in aqueous solution and morphological changes with time were followed by using transmission electron microscopy. During this time frame under the utilized conditions potential signs of degradation were visible (see Figure 5), promising as a preliminary proof-of-concept, yet not conclusive, of using degradable phosphazene bridges within the silica framework.

To enhance their degradation, linear polyphosphazenes with different side groups could be utilized in future work for the preparation of silica-based microparticles, thus, the degradation rate could potentially be accelerated. Through the choice of the side groups, the degradation rate may be tuned, thus if more hydrophilic groups are used, the faster the material is expected to degrade [29]. Such novel phosphazene containing organosilica materials might be used, for example for gene delivery, due to their structure containing large pores [30].

### 3.5. Post-Functionalization of the Porous SiCPz2-PM2 Microparticles

**Figure 6:**
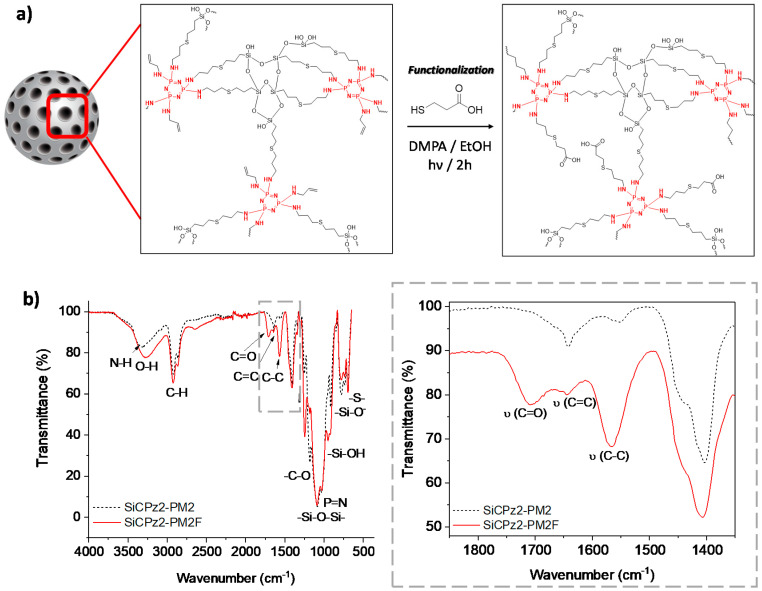
(**a**) Post-functionalization scheme of the porous organosilica microparticles SiCPz2-PM2 with 3-mercaptopropionic acid yielding to functionalized microparticles SiCPz2-PM2F. (**b**) FT-IR spectra of the porous organosilica microparticles before and after functionalization (SiCPz2-PM2 and SiCPz2-PM2F, respectively) and zoom-in from 1850 to 1350 cm^−1^ showing the most characteristics functionalized groups.

Functionalization of mesoporous silica particles is of interest for some advanced applications, for example for carrying drug molecules [31]. The potential post-functionalization features of this type of novel porous microparticles was demonstrated by using SiCPz2-PM2. The double bonds present in these particles were post-functionalized with 3-mercaptopropionic acid using the same thiol-ene photoreaction utilizing again DMPA as initiator (adapted from [32]), yielding in the microparticles SiCPz2-PM2F as seen in Figure 6a. The successful functionalization of the particles was checked by both, thermogravimetric analyses (Figure S10) and FT-IR measurements (see Figure 6b). From the thermogravimetric studies of SiCPz2-PM2F, an increased organic content difference of ca. 10 % in comparison to the pristine material SiCPz2-PM2 was determined.

FT-IR spectroscopy was used to confirm the functionalization of the SiCPz2-PM2 particles with 3-mercaptopropionic acid. Mainly, this was achieved by detecting the appearance of the carbonyl (C=O) peak at 1708 cm^−1^ along with the increase of the broad peak at 3285 cm^−1^ from the –OH groups in the spectrum of SiCPz2-PM2F (see Figure 6b). Interestingly, the peak at 1643 cm^−1^ assigned to the free C=C functional groups decreased after UV light irradiation, potentially indicating the occurrence of an additional click reaction between 3-mercaptopropionic acid and allylamine, attaching the carboxylic acid group to the terminal double bonds of the allylamine chains.

## 4. Conclusions

Novel hybrid porous phosphazene-containing organosilica materials were prepared using a surfactant mediated sol-gel method, resulting in porous spherical microparticles. The phosphazene precursor was prepared in a two-step reaction. In the first step, allylamine was used as substitution reagent to replace the chlorine atoms of the cyclic phosphazene trimer. In the second step, the silane containing (3-mercaptopropyl)trimethoxysilane was linked to the double bonds via a thiol-ene photoreaction. After the thiol-ene reaction, two different precursors were obtained, a fully substituted one (all double bonds were reacted with the thiol, SiCPz1) and a half-substituted one (SiCPz2), which had double bonds left, useable for further post-functionalization. These phosphazene precursor solutions were then directly used for the microparticle synthesis, yielding to SiCPz1-PM1 and SiCPz2-PM2. In both cases, spherical microparticles (with an average diameter of around 1000 nm) and large pores (from around 70 to 250 nm) were obtained. An effect on the size distribution related to the number of reactive alkoxysilanes was observed, whereby using a phosphazene-based precursor with a larger amount of reactive silane groups may lead to a faster particle formation from the beginning, and thus to a more homogeneous particle size distribution. Furthermore, hydrolytic degradation studies were performed using the SiCPz1-PM1 for 4 weeks in an aqueous solution at pH 2 and pH 7 observing a potential, not yet conclusive degradable behavior. The remaining double bonds of the SiCPz2-PM2 porous organosilica microparticles were further able to be functionalized using a thiol-ended molecule via UV-photoreaction. This thus represents a promising new method for the preparation of porous phosphazene-based silica microparticles which could be of interest for a range of applications, such as drug delivery, adsorbents, and sensors.

## Supplementary Materials

The following are available online at https://www.mdpi.com/1422-0067/21/22/8552/s1.
