# Supplementary material for: Hybrid Porous Microparticles Based on a Single Organosilica Cyclophosphazene Precursor
Source: Int J Mol Sci. 2020 Nov 13;21(22):8552. doi: 10.3390/ijms21228552 (PMC7698118; doi:10.3390/ijms21228552)
Supplement: Supplementary file 1 [file ijms-21-08552-s001.pdf]

Supporting Information

Hybrid porous microparticles based on a single organosilica cyclophosphazene precursor

Vanessa Poscher, [1,2] *George S. Pappas,*[1] *Oliver Brüggemann,*[1] *Ian Teasdale*[1,2]

and Yolanda Salinas.[1,2]*

[1] Institute of Polymer Chemistry (ICP), Johannes Kepler University Linz, Altenberger Strasse 69, 4040 Linz, Austria.

[2] Linz Institute of Technology (LIT), Johannes Kepler University Linz, Altenberger Strasse 69, 4040 Linz, Austria.

Corresponding author:

E-mail: yolanda.salinas@jku.at

Characterization of the silane derived cyclic phosphazenes, precursors SiCPz1-2.

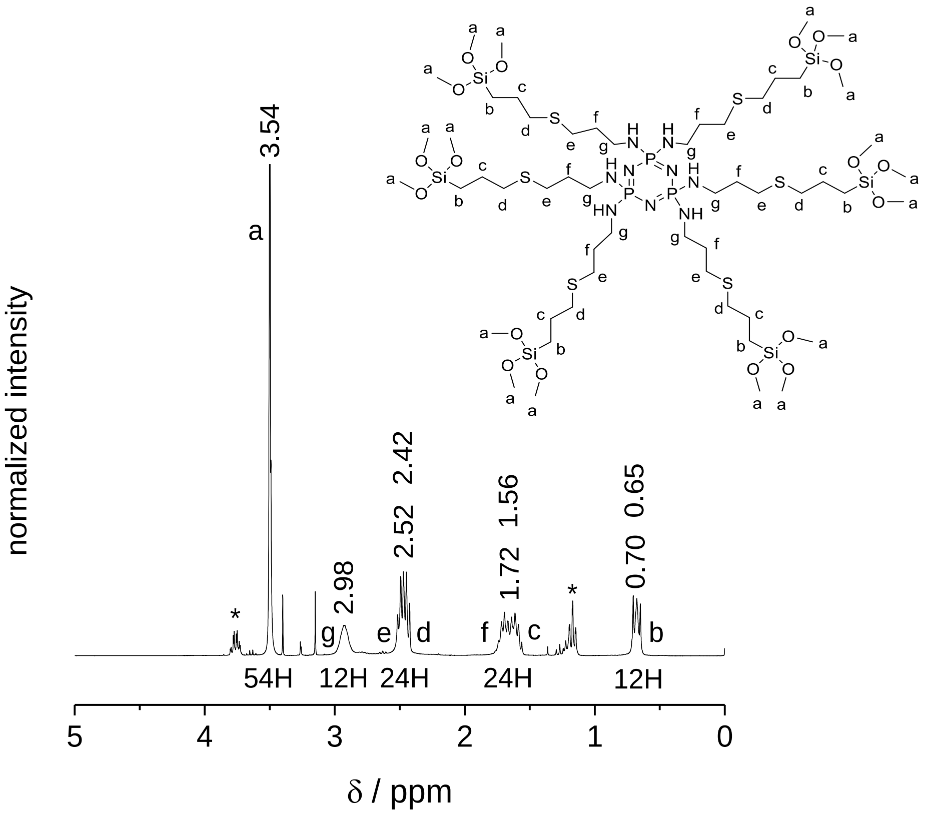

Figure S1. ^{1}H NMR spectrum of fully substituted precursor SiCPz1 in $CDCl_3$ (EtOH marked as *), synthesis of this molecule previously reported by our group [1].

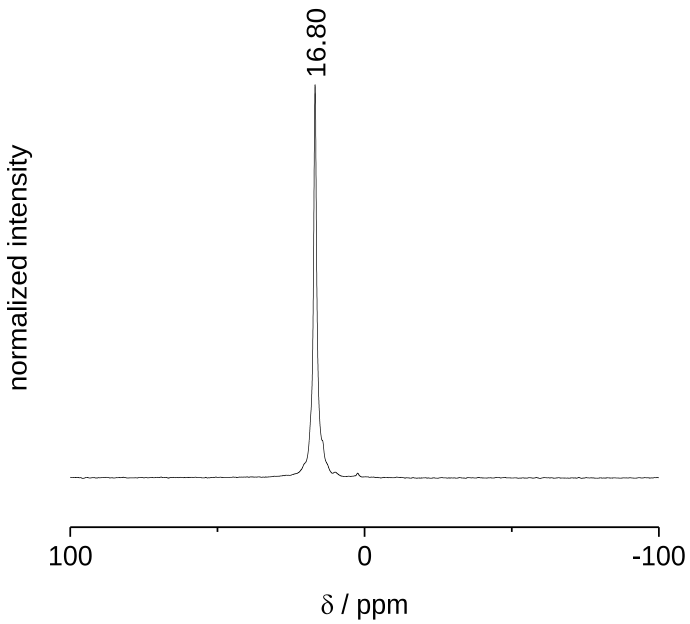

Figure S2. ^{31}P NMR spectrum of fully substituted precursor SiCPz1 in $CDCl_3$.

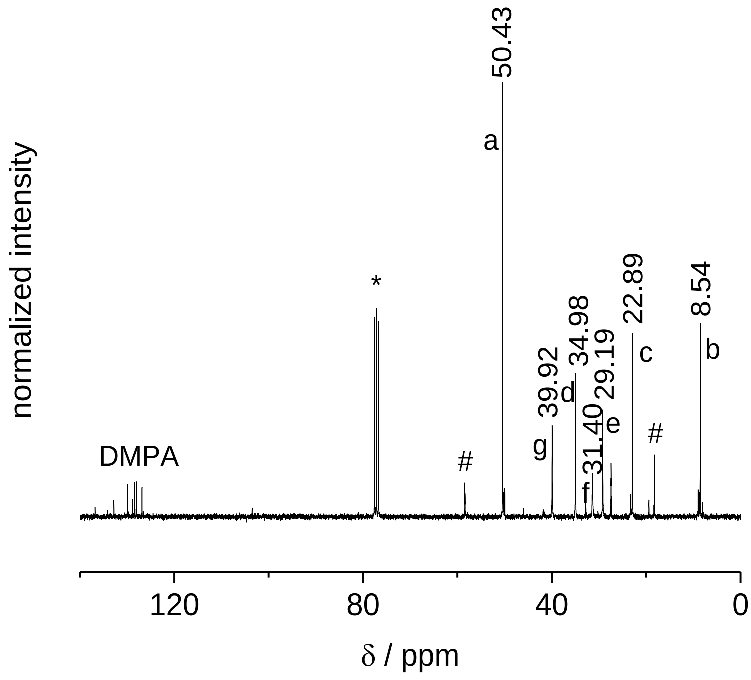

Figure S3. ^{13}C NMR spectrum of fully substituted precursor SiCPz1 in $CDCl_3$ (*), EtOH (#).

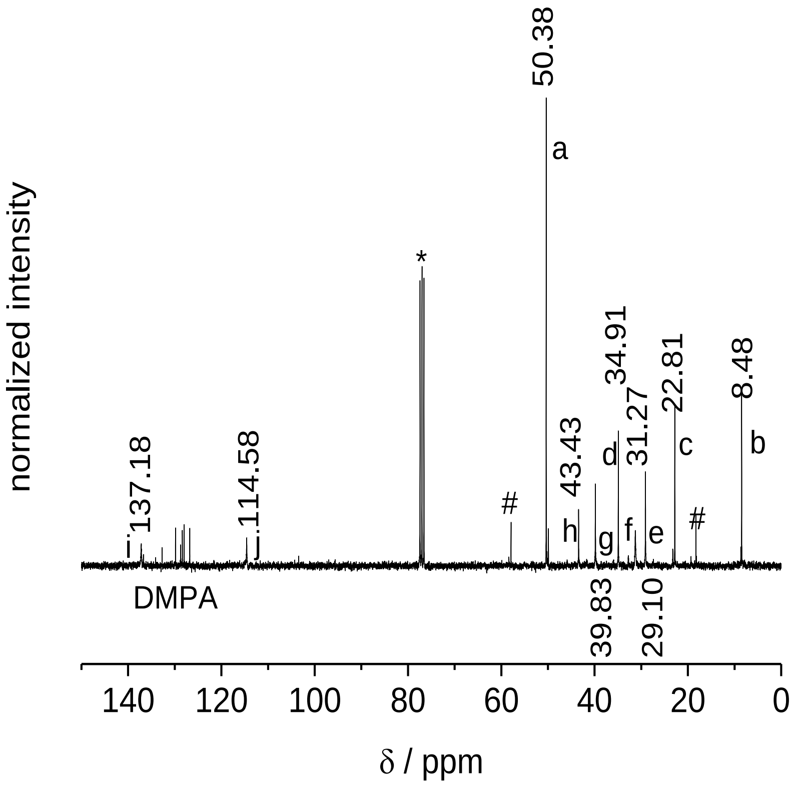

Figure S4. ^{13}C NMR spectrum of half-substituted precursor SiCPz2 in $CDCl_3$ (*), EtOH (#).

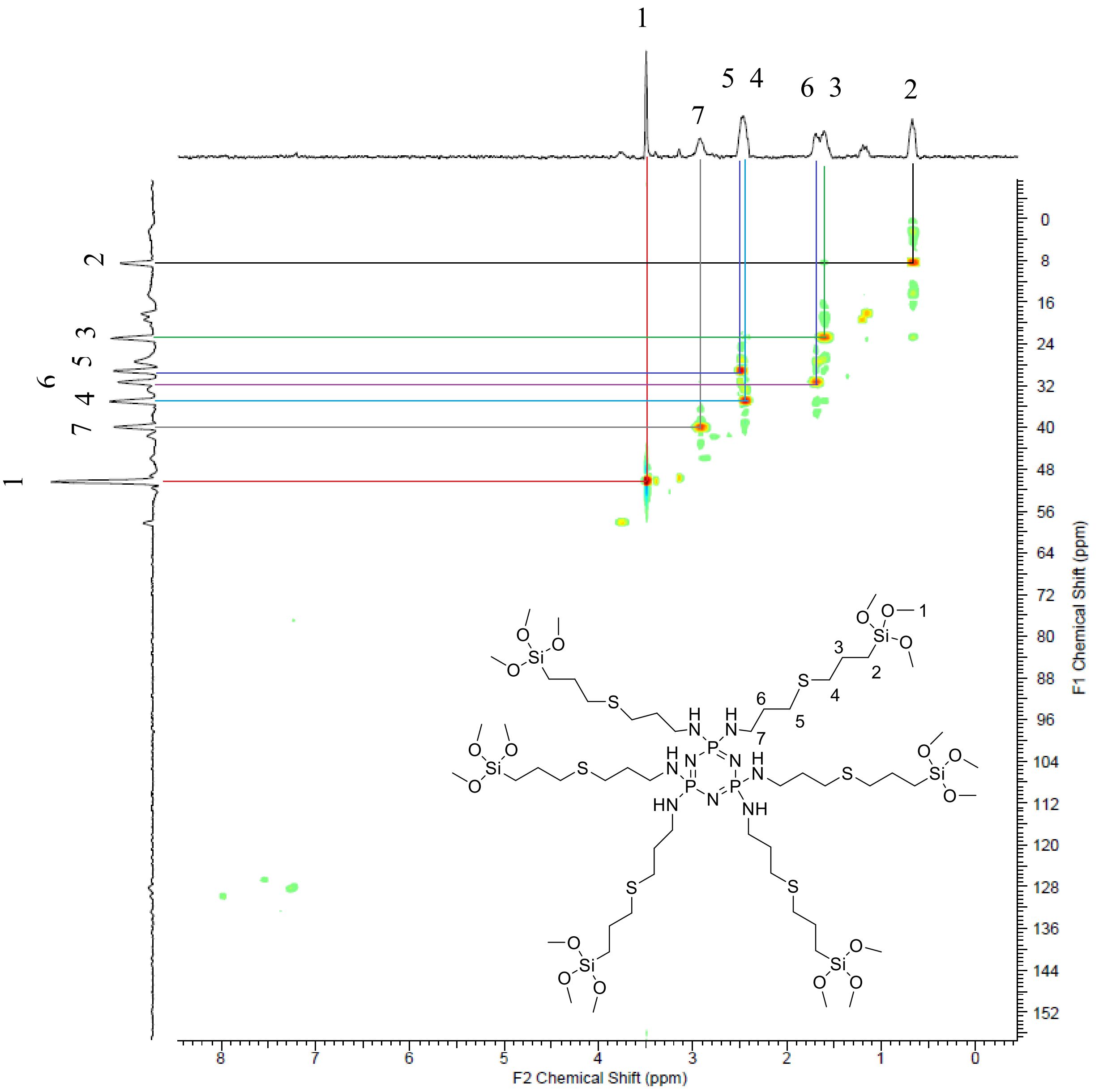

Figure S5. HSQC spectrum of the fully substituted precursor SiCPz1.

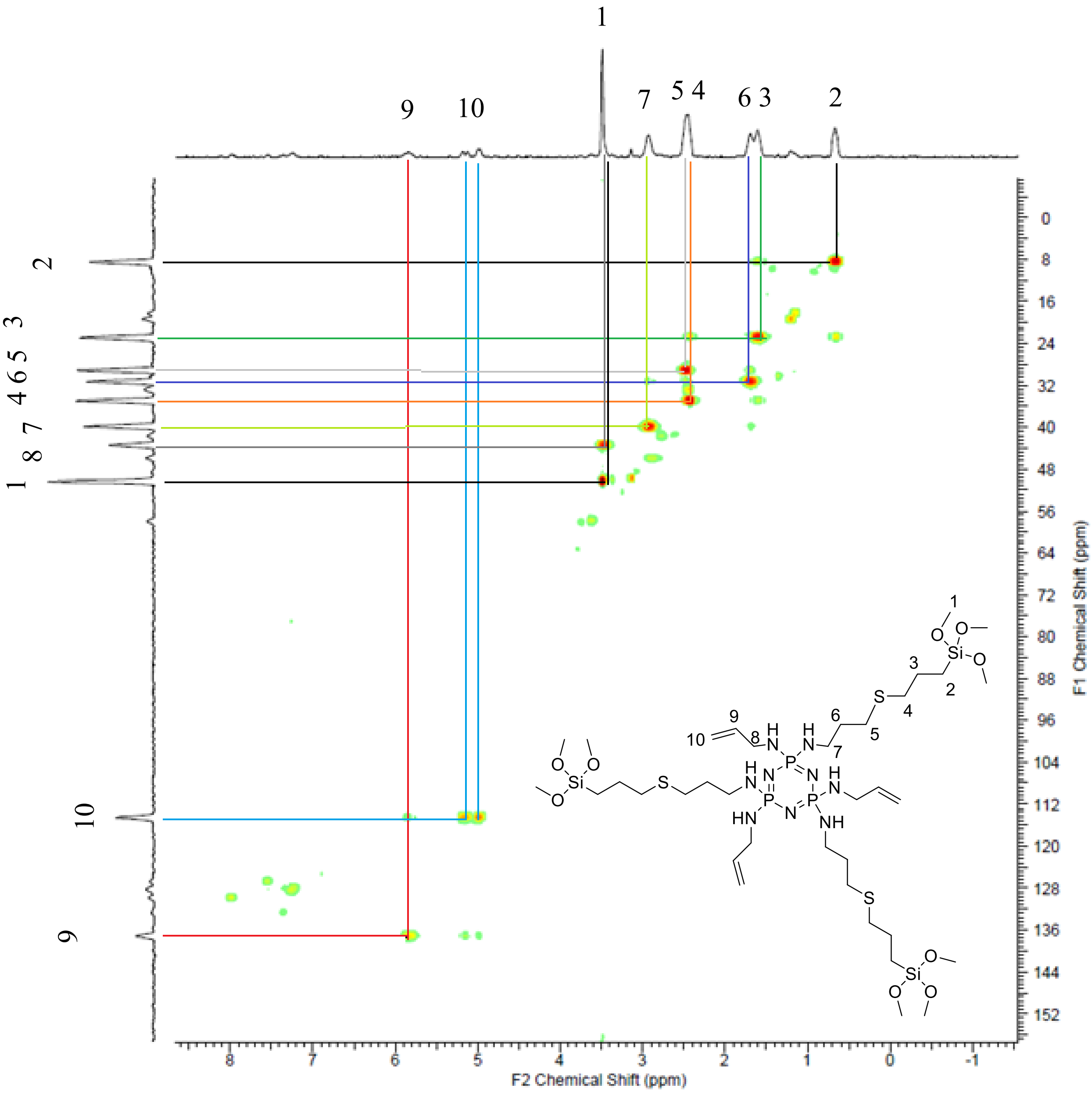

Figure S6. HSQC spectrum of the half-substituted precursor SiCPz2.

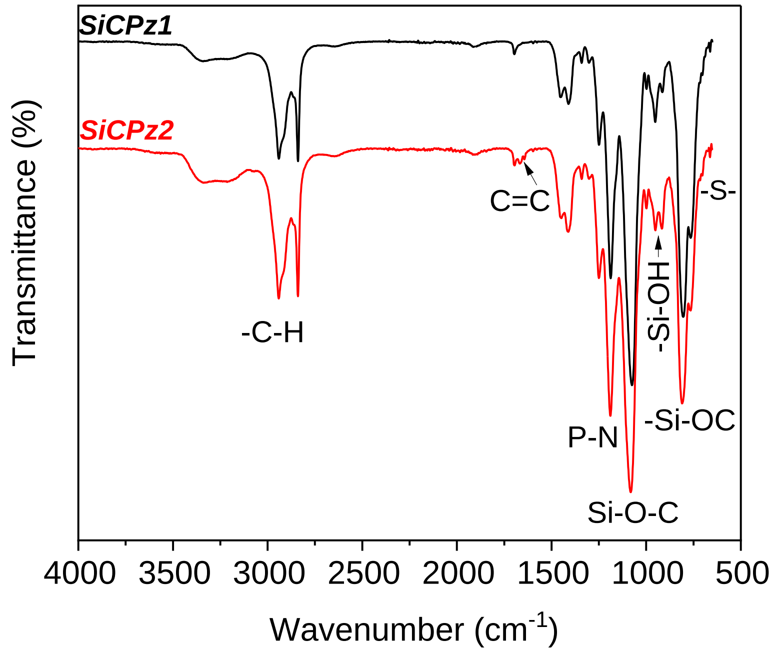

Figure S7. FT-IR spectra of fully and half-substituted precursors SiCPz1 and SiCPz2.

Porous organosilica microparticles characterization.

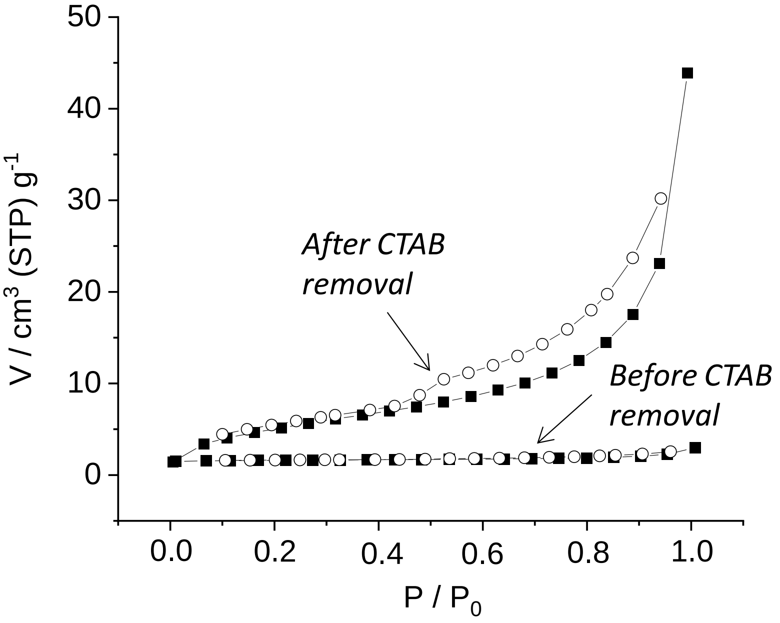

Figure S8. Nitrogen adsorption-desorption isotherms of SiCPz2-PM2 porous microparticles before and after surfactant (CTAB) removal. Black squares are assigned to the adsorption and white circles assigned to the desorption process.

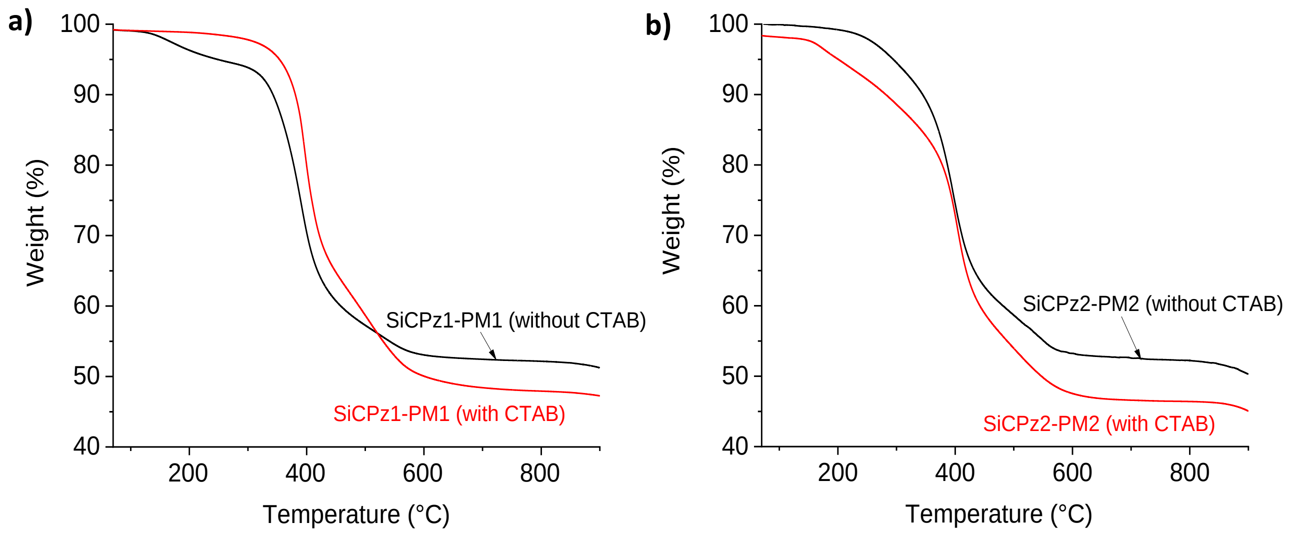

Figure S9. Thermogravimetric analysis of a) SiCPz1-PM1 and b) SiCPz2-PM2 microparticles before (with CTAB, red line) and after surfactant removal (without CTAB, black line), under nitrogen atmosphere in a platinum pan, from 50 to 900 °C.

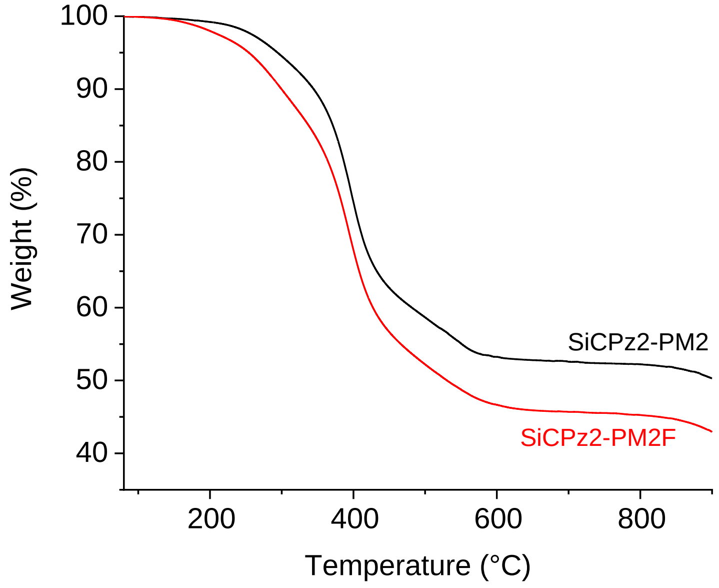

Figure S10. Thermogravimetric analysis before (SiCPz2-PM2, black line) and after functionalization with 3-mercaptopropionic acid (SiCPz2-PM2F, red line).

References

[1] Poscher, V.; Teasdale, I.; Salinas, Y. Surfactant-Free Synthesis of Cyclomatrix and Linear Organosilica Phosphazene-Based Hybrid Nanoparticles. *ACS Appl. Nano Mater*. **2019**, *2*, 655-660.
